# Associations of Alcohol and Tobacco Retail Outlet Rates with Neighborhood Disadvantage

**DOI:** 10.3390/ijerph19031134

**Published:** 2022-01-20

**Authors:** David C. Wheeler, Joseph Boyle, D. Jeremy Barsell, Trevin Glasgow, F. Joseph McClernon, Jason A. Oliver, Bernard F. Fuemmeler

**Affiliations:** 1 Department of Biostatistics, Virginia Commonwealth University, Richmond, VA 23298, USA; boylejr@vcu.edu; 2 Department of Health Behavior and Policy, Virginia Commonwealth University, Richmond, VA 23298, USA; barselldtj@vcu.edu (D.J.B.); trevin.glasgow@vcuhealth.org (T.G.); Bernard.fuemmeler@vcuhealth.org (B.F.F.); 3 Department of Psychiatry and Behavioral Sciences, Duke University School of Medicine, Durham, NC 27705, USA; francis.mcclernon@duke.edu (F.J.M.); jason-oliver@ouhsc.edu (J.A.O.); 4 Stephenson Cancer Center, University of Oklahoma Health Sciences Center, Oklahoma City, OK 73104, USA; 5 Department of Psychiatry and Behavioral Sciences, Oklahoma State University Center for Health Sciences, Tulsa, OK 74107, USA; 6 Massey Cancer Center, Virginia Commonwealth University, Richmond, VA 23298, USA

**Keywords:** alcohol, tobacco, retail outlets, neighborhood disadvantage, neighborhood deprivation

## Abstract

Tobacco causes 29% of cancer-related deaths while alcohol causes 5.5% of cancer-related deaths. Reducing the consumption of these cancer-causing products is a special priority area for the National Cancer Institute. While many factors are linked to tobacco and alcohol use, the placement and density of retail outlets within neighborhoods may be one community-level risk factor contributing to greater use of these products. To elucidate associations between tobacco, alcohol, and tobacco and alcohol retail outlets (TRO, ARO, and TARO) and neighborhood disadvantage over a large geographic area, we employed a novel Bayesian index modeling approach to estimate a neighborhood disadvantage index (NDI) and its associations with rates of the three types of retailers across block groups in the state of North Carolina. We used a novel extension of the Bayesian index model to include a shared component for the spatial pattern common to all three types of outlets and NDI effects that varied by outlet type. The shared component identifies areas that are elevated in risk for all outlets. The results showed significant positive associations between neighborhood disadvantage and TROs (relative risk (RR) = 1.12, 95% credible interval (CI = 1.09, 1.14)) and AROs (RR = 1.15, 95% CI = 1.11, 1.17), but the association was greatest for TAROs (RR = 1.21, 95% CI = 1.18, 1.24). The most important variables in the NDI were percent renters (i.e., low home ownership), percent of homes built before 1940 (i.e., old housing stock), and percent without a high school diploma (i.e., low education).

## 1. Introduction

Reducing the consumption and access of cancer-causing products, such as alcohol and tobacco, is of high public health interest. Tobacco use causes 29% and alcohol use causes 5.5% of cancer-related deaths [1]. Alcohol-attributed female cancer deaths are primarily from breast cancer (55–66%), whereas alcohol-attributed cancers in men are primarily of the aero-digestive tract (53–71%) [2]. The prevalence of heavy alcohol use (i.e., diagnosed with an alcohol use disorder) in the US is 7% among men and 4% among women [3], whereas the smoking prevalence rates are 15.3% among men and 12.7% among women [4]. Additionally, youth in the US are at risk of using these products. For example, e-cigarette use is high among youth, with 19% of high school students reporting using e-cigarettes over the past 30 days [5,6].

While there are a number of factors linked to tobacco and alcohol use (e.g., peer pressure, genetics, and personality), the placement and density of alcohol and tobacco retail outlets within neighborhoods may be one community-level risk factor contributing to greater use of these products. State and local communities have the ability to regulate both alcohol and tobacco retail through licensing and other policies such as zoning. Presumably, reducing the density of these retail outlets has the potential to facilitate change in behavior of community members by altering the socio-spatial contexts leading to alcohol and tobacco use. This likely happens by reducing residents’ access and exposure to the products and product-related marketing but also by disrupting the “collective lifestyle” or social practices supportive of use (i.e., social norms about acceptability of use) [7,8,9]. To inform such efforts, a number of studies have examined (1) retail density in relationship to socio-spatial demographic predictors of health disparities to better understand where these retail environments cluster [10,11] and (2) the extent to which retail density might be linked to increased consumption of cancer-causing products [12]. With some caveats, findings from these studies reveal that both tobacco retail outlet (TRO) and alcohol retail outlet (ARO) density are greater in neighborhoods with lower median income households and higher percent minority residents [13,14]. However, few studies have examined the intersection of TROs and AROs and the extent to which they correlate with other socio-economic characteristics of the neighborhood environment. The literature generally supports that greater density of these retail outlets is associated with greater use of tobacco and alcohol products among those living within these neighborhoods [15,16].

The purpose of this study was to better understand the extent to which neighborhood socio-demographic characteristics relate to both the overlapping and distinct spatial distributions of tobacco, alcohol, and tobacco and alcohol retail outlets (TRO, ARO, and TAROs). To do so, we employed a Bayesian index model approach [13,17] with new extensions to estimate a neighborhood disadvantage index (NDI) and its associations with rates of these three types of retail outlets across the state of North Carolina at the block group level. We used a novel extension of the Bayesian index model to include a shared component for the spatial pattern common to all three types of outlets and NDI effects that varied by outlet type. The shared component is used to identify areas that are elevated in risk for all three outlet types. We hypothesized that higher NDI as measured by a weighted combination of neighborhood socioeconomic status (SES) indicators (e.g., education, income, and poverty) and racial/ethnic segregation (e.g., black population segregation) would be associated with higher rates of each of the three types of outlets, but that the association may differ for each outlet type. We also sought to identify the geographic areas that were significantly elevated in risk for each of the three outlet types.

## 2. Materials and Methods

### 2.1. Study Context 

The study context for these analyses was the state of North Carolina. North Carolina does not require retailers to have a license to sell tobacco but does regulate the sale of some alcohol products. The North Carolina Alcoholic Beverage Control (ABC) regulates alcohol sales and permits retailers to sell beer and wine, but liquor can only be purchased in a state-run ABC store. The ABC Commission considers a number of factors when issuing retail beer and wine permits, such as the number of existing ABC permits within a neighborhood, parking, traffic, the kinds of businesses already in the neighborhood, and proximity to schools and churches [18].

### 2.2. Study Data

#### Tobacco and Alcohol Retail Outlets

We obtained data for the three outlet types from two different sources. The first database we used was the National Establishment Time Series (NETS), a private, longitudinal, and geocoded record of businesses of all types in the United States. We utilized a previously developed algorithm to identify TROs [19]. The algorithm filtered entries in the database by their North American Industry Classification System codes to only include TROs, deleted duplicate records, and removed records for TROs in years after state- or municipality-specific bans were enacted on the sale of tobacco products by certain business types. We sourced ARO data from the North Carolina ABC Commission. The state’s ABC maintains a publicly available database [18] of businesses that have been granted licenses to sell alcoholic beverages, including ABC liquor stores. We pulled the business entries from this database that had active and off-premise licenses to sell alcohol to avoid including businesses such as bars and restaurants. We geocoded AROs from their business address using the ESRI software (ESRI, Redlands, CA, USA) ArcMap and then standardized several features of the data to maximize similarity between the TRO and ARO databases, including converting all text to upper case, retaining a common street suffix, and removing store identification numbers from business names. We included tobacco and alcohol retail outlets that were stated to be active any time in the year 2019. 

Our process to count the number of TROs, AROs, and TAROs in each block group in North Carolina began with intersecting the outlets with block group boundaries. We used the 2017 TIGER/Line Shapefiles from the U.S. Census Bureau for block group boundaries [20]. Then, we identified TAROs from a list of TROs and AROs for each block group. As a first step, we populated a list of the approximately 100 most commonly appearing businesses from the NETS database that were known TAROs, including major gas stations and grocery stores (e.g., “CIRCLE K STORES INC” and “FOOD LION LLC”). If a block group contained only TROs or only AROs, we assigned its TARO count to be the cardinality of the set intersection of its business listings and the list of commonly known TAROs. This step was necessary due to a potential for the business listings to incorrectly exclude listings that existed in the block group. For example, if a block group had a popular grocery store in its ARO list, but no business in its TRO list, then this step would catch the fact that the grocery store was a TARO. If the block group contained at least one TRO and at least one ARO, we used a custom similarity score to assign its TARO count. Specifically, for each TRO in the block group, we compared it to each ARO in the block group with respect to business name, address, and spatial coordinates (latitude and longitude). We calculated the string similarity between the business names and addresses using optimal string alignment, which is a restricted measure of the Damerau–Levenshtein distance [21]. The distance between two strings was divided by the maximum possible distance between them and subtracted from one, so that more similar strings had higher scores and scores were bounded by [0,1]. We calculated the spatial similarity between each TRO and ARO using two thresholds: one-tenth and one-twentieth of a mile. We used these thresholds to allow for the possibility of error in address records or in geo-coding and to give higher likelihoods of similarity to businesses that were very close to each other.

The total similarity between any TRO listing *t* and ARO listing *a* was calculated based on name similarity, address similarity, and spatial similarity as

(1) $$\begin{array}{ll} sim\left(t,a\right)=0.25 & *\left(namesim\left(t,a\right)\right)+0.25*\left(addresssim\left(t,a\right)\right)+0.20\\ & *\left(I\left(dist\left(t,a\right)<520.9ft\right)\right)+0.30\\ & *\left(I\left(dist\left(t,a\right)<260.45ft\right)\right), \end{array}$$

where the distances in feet represent one-tenth and one-twentieth of a mile, respectively, I(⋅) denotes the indicator function, and dist(⋅) denotes the great circle distance between two locations. The component weights were chosen to prioritize spatial similarity, as businesses in the two databases that were extremely close to each other were more likely to be the same entity. A total similarity score of greater than or equal to 0.70 was deemed a match, and these businesses were considered to be one TARO and were removed from the lists of TROs and AROs to compare. We chose the threshold value of 0.70 following a sensitivity analysis that manually checked the results of the similarities for several block groups and found it to differentiate matches from non-matches with high sensitivity and specificity. At the end of this process, each block group had a count of businesses that were just TROs, just AROs, or TAROs. Across all the block groups in the state, we identified 7289 TROs, 4859 AROs, and 4631 TAROs.

### 2.3. Sociodemographic Data

We used five-year (2014–2018) estimates of nine sociodemographic variables at the census block group level from the American Community Survey (ACS) to construct neighborhood disadvantage indices. The ACS is administered annually to three million households, representative of the US population. Participants complete a questionnaire and report their household’s social and economic information. We chose this time period to accord with the tobacco and alcohol outlet data. The sociodemographic variables were: Black population segregation, Hispanic population segregation, percent with ratio of income to poverty level <1, percent households with public assistance income, percent renter occupied housing units, percent homes built 1939 or earlier, percent with no high school degree or higher, percent of household in poverty, and per capita income. This set of variables covers several dimensions of the racial and socio-economic composition of the block groups. For example, the percent of homes built 1939 or earlier is a proxy for the quality of the housing stock in that block group. Black segregation was defined as the percent of population that is Black in a block group divided by the state average percent Black population. Hispanic segregation was similarly defined. We have used similar sociodemographic variables to estimate neighborhood disadvantage indices previously [13,17]. We started with 6127 block groups for the state and removed 15 due to having no population and 29 others due to missing covariates to reach an analysis set of 6083. In this set of block groups, there were a total of 7279 TROs, 4849 AROs, and 4621 TAROs.

### 2.4. Statistical Analysis

We used Bayesian hierarchical regression models to explain the variation in the rates of the three outlet types, assuming that the count of the *k*th outlet type (e.g., TRO, ARO, or TARO) in the *i*th block group was $y_{ik}\sim Poisson\left(\theta_{ik}E_{ik}\right)$ with relative risk $\theta_{ik}$ and expected count $E_{ik}$, where the expected count for each block group was calculated as the overall outlet type rate $r_{k}$ multiplied by the population $p_{i}$ in the block group. The Bayesian shared component index model of the log relative risk was

(2) $$\log(\theta_{ik})=\alpha_{k}+\beta_{k}\left(\sum_{j=1}^{C}w_{j}q_{ij}\right)+u_{i}+v_{i}+s_{ik}$$

where $\alpha_{k}$ is the outlet-specific intercept, $\beta_{k}$ is the outlet-specific effect for the neighborhood disadvantage index, $u_{i}$ is a shared component that is spatially random, $v_{i}$ is a shared component that is spatially correlated, and $s_{ik}$ is an outlet-specific component that is spatially correlated. The model as specified has a shared risk component for the three outlet types as well as an outlet-specific component for risk. The total shared component of relative risk among the three outlet types for each block group was $\exp(u_{i}+v_{i})$. The term $\exp(s_{ik})$ is the component of relative risk for each block group that is specific to each outlet type. 

We specified the neighborhood deprivation index for each block group using a weighted combination $\overset{C}{\underset{j=1}{\sum }}w_{j}q_{j}$ of the deciles $q_{1},\ldots ,q_{c}$ of the sociodemographic variables $x_{1},\ldots ,x_{c}$, where the weights $w_{1},\ldots ,w_{C}$ were estimated in the model. The weight $w_{j}$ represents the relative importance of the $j_{th}$ variable in the index. We used deciles of the variables to account for different scaling of the variables, de-correlate the variables, limit the effect of outliers, and acknowledge uncertainty in the ACS values. We used C=9 variables in the index. The sociodemographic variables were defined to reflect a hypothesized positive association of the index with relative risk (i.e., higher disadvantage associated with greater relative risk). Per capita income was redefined to have a positive association with rates based on univariate correlations with the outlet rates. We inverted this variable by using the formula $\max\left(x\right)-x_{j}$, where $x_{j}$ is the value of the variable. 

The Bayesian model specification was completed with the definition of prior distributions for the priors. The index weights were given a Dirichlet prior with parameters $\alpha =\left(\alpha_{1},\ldots ,\alpha_{C}\right)$. The Dirichlet prior was used because it assures that the index weights $w_{j}\in \left(0,1\right)$ and $\overset{C}{\underset{j=1}{\sum }}w_{j}=1$. The intercepts followed an improper uniform distribution $\alpha_{k}\sim dflat()$, while the index regression coefficients had a vague normal prior $\beta_{k}\sim Normal\left(1,\tau_{\beta }\right)$ with precision $\tau_{\beta }=1/\sigma_{\beta }^{2}$ and $\sigma_{\beta }\sim Uniform\left(0,100\right)$. The spatially correlated components $v_{i}$ and $s_{ik}$ received intrinsic conditional autoregressive (CAR) priors, $v_{i}\sim CAR(\tau_{v})$ and $s_{ik}\sim CAR(\tau_{k})$ with priors for the precisions of $\tau_{v}\sim Gamma(0.05,0.005)$ and $\tau_{k}\sim Gamma(0.05,0.005)$. 

We used Markov chain Monte Carlo (MCMC) to estimate the model parameters with a total of 75,000 iterations from two chains and a thinning parameter of one, where the first 60,000 iterations were used for burn-in. We assessed the convergence of the MCMC algorithm for parameters of interest using the Gelman–Rubin convergence diagnostic [22]. A parameter was considered to have converged if its diagnostic absolute value was less than 1.2. See supplemental material Figure S1 for diagnostic plots of key model parameters. The 95% credible interval was used to determine the statistical significance of the disadvantage index, with there being a significant effect if the interval did not contain the value of 1. Exceedance probabilities were used to identify block groups with significantly elevated relative risk of each outlet type using a relative risk threshold of 1 and a threshold probability of 0.90. In addition, we calculated the mean relative risk and percent of significantly elevated relative risks by population density category for each outlet type, where population density categories were rural (<500 person/square mile), suburban (≥500 and <1000 persons/square mile), and urban (≥1000 persons/square mile). We fit the Bayesian models using R2OpenBUGS [23] in the R computing environment [24].

## 3. Results

**Figure 1:**
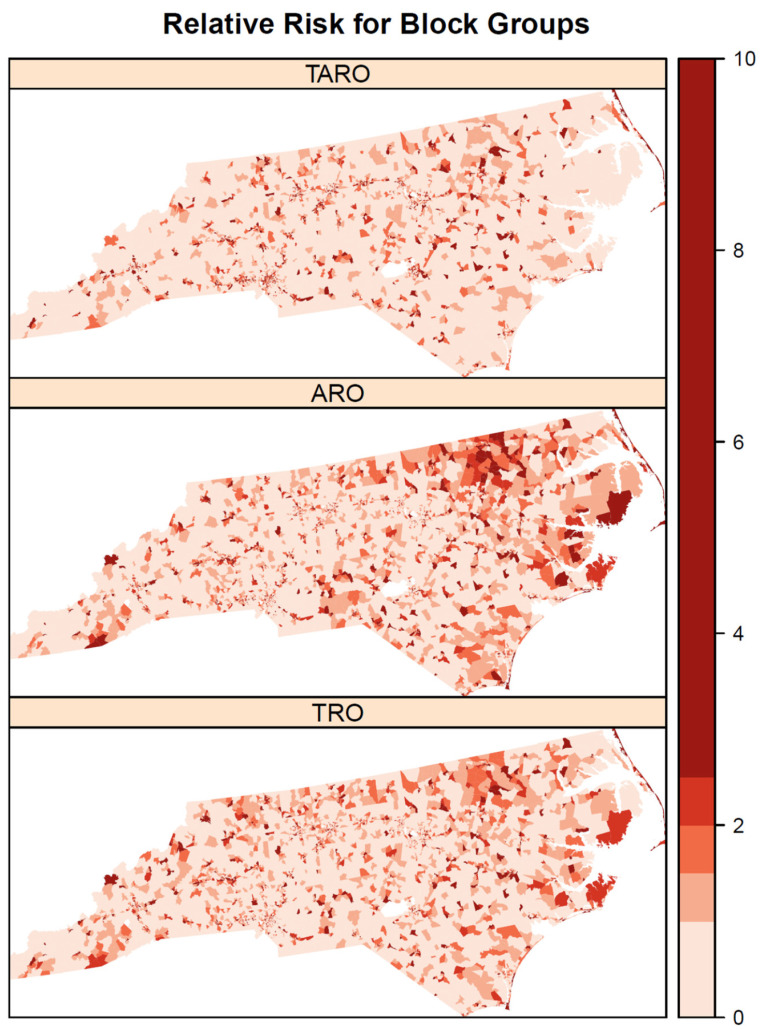
Relative risk for tobacco retail outlet (TRO), alcohol retail outlet (ARO), and tobacco and alcohol retail outlet (TARO) modeled from Bayesian shared component index model.

**Figure 2:**
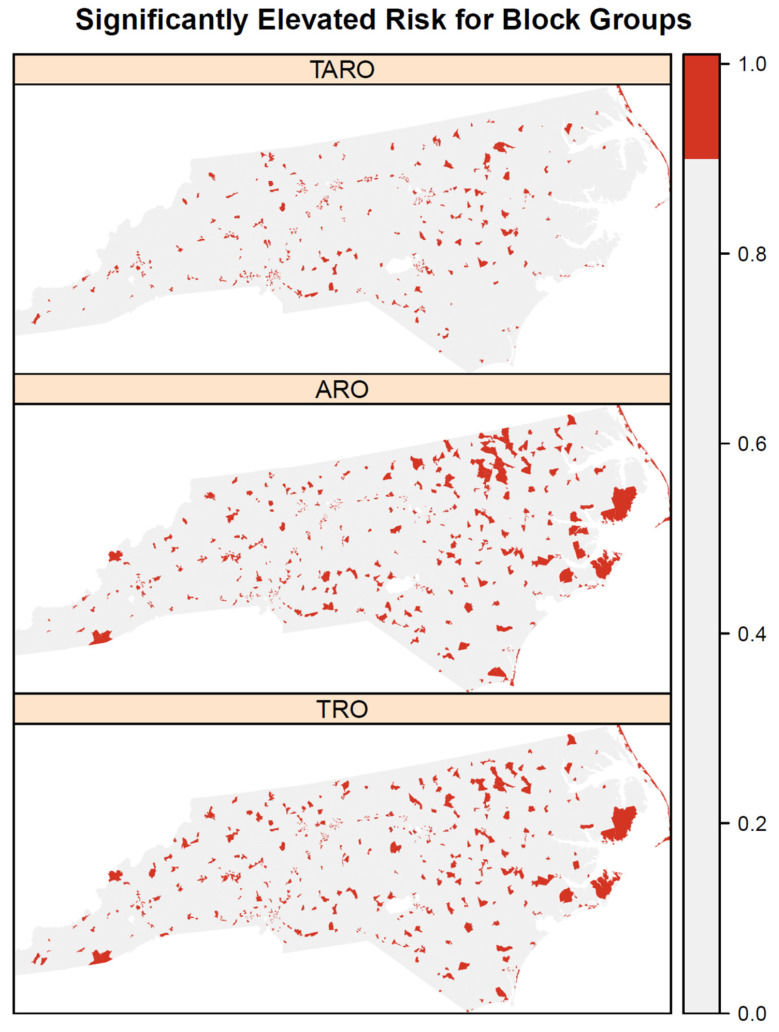
Block groups with significantly elevated relative risk for tobacco retail outlet (TRO), alcohol retail outlet (ARO), and tobacco and alcohol retail outlet (TARO) based on exceedance probabilities.

Among the three types of outlets, the pairwise correlations in the observed rates were 0.41 for TROs and AROs, 0.51 for TROs and TAROs, and 0.20 for AROs and TAROs. Some correlations between outlet types are also observed in the modeled relative risks (Figure 1), where relative risks are high for all outlet types in the northeastern coastal area. Areas of high relative risk for TROs and AROs are evident in northeastern North Carolina and the central coastal area. All three outlet types also have areas of elevated risk in southwestern North Carolina. The patterns of high risk are confirmed when using exceedance probabilities of the relative risk to identify significantly elevated risk areas (Figure 2). The northeastern coastal area is significantly elevated in risk for all three outlet types and several areas are identified for both AROs and TROs in the northeast and southeastern coastal area. In contrast, the areas of significantly elevated risk for TAROs are smaller and less clustered. According to the mean relative risks and percent of block groups with significantly elevated relative risks, suburban areas were at greatest risk of having tobacco, alcohol, and tobacco and alcohol outlets, followed by urban areas and then rural areas (Table 1). The highest mean relative risks were for AROs (1.44) and TAROs (1.44) in suburban areas, whereas the largest percent of significantly elevated relative risks was for TROs (0.20) followed by AROs (0.19) in suburban areas.

**Figure 3:**
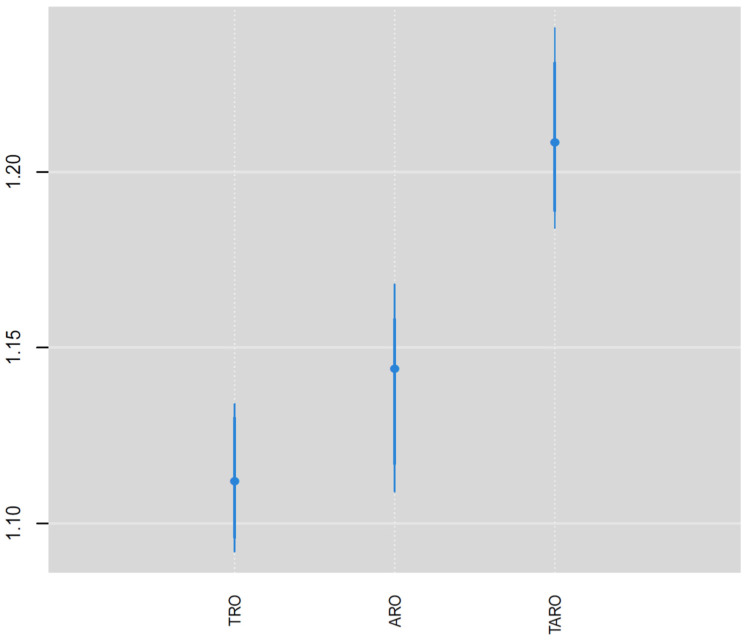
Posterior medians and 95% credible intervals for neighborhood disadvantage index effects by outlet type.

**Figure 4:**
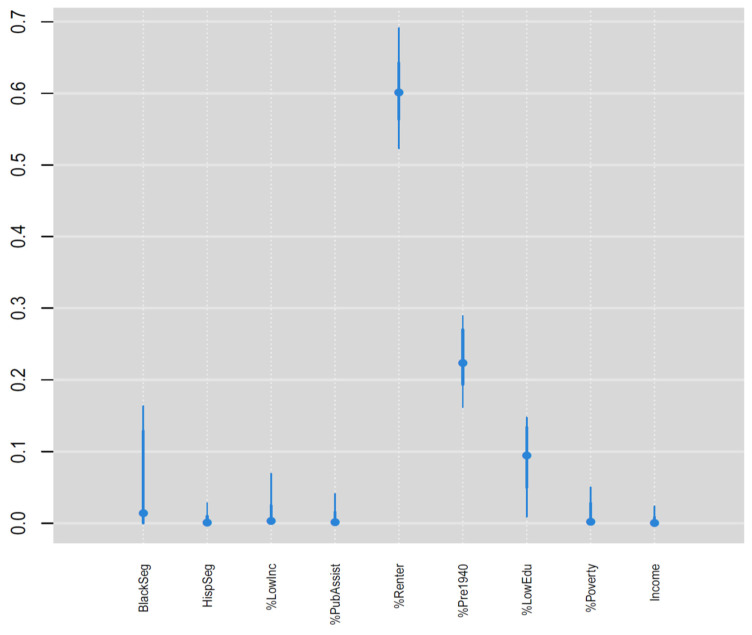
Posterior medians and 95% credible intervals for the neighborhood disadvantage index weights.

**Figure 5:**
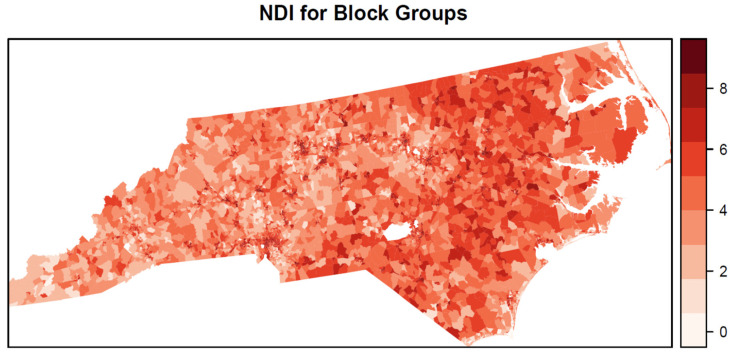
Neighborhood disadvantage index for block groups estimated from the Bayesian shared component index model.

The relative risk of the NDI varied by outlet type (Figure 3), where it was highest for TAROs (1.21) followed by AROs (1.15) and then TROs (1.12). All three positive NDI effects are significant according to the 95% credible intervals exceeding relative risk values of 1. The relative risk was significantly higher for TAROs compared with AROs and TROs according to the 95% credible intervals (Figure 3), whereas the credible intervals substantially overlapped for TROs and AROs. The weights for the nine components in the NDI (Figure 4) show that percent renter (0.60), percent of homes built before 1940 (0.23), and percent without a high school degree (0.09) were the three most important variables in the NDI and together accounted for 0.92 of the total weight. Aside from black segregation (0.03), all other weights were less than 0.02. The estimated NDI has a greater concentration of higher values in the eastern half of the state compared with the western half (Figure 5). However, clusters of high neighborhood deprivation are evident in metropolitan areas in the center and western half of the state.

**Figure 6:**
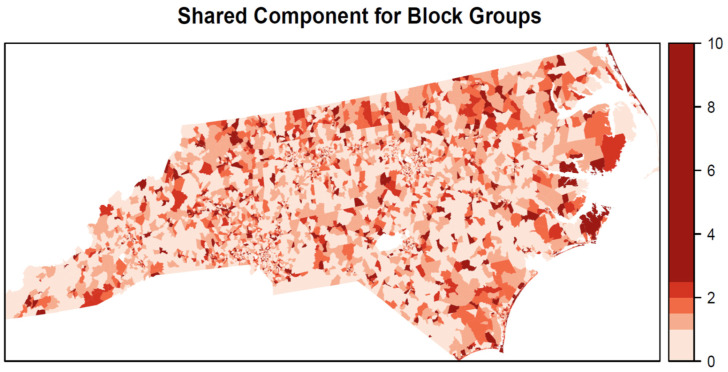
Shared risk component among the three outlet types (TRO, ARO, and TARO).

**Figure 7:**
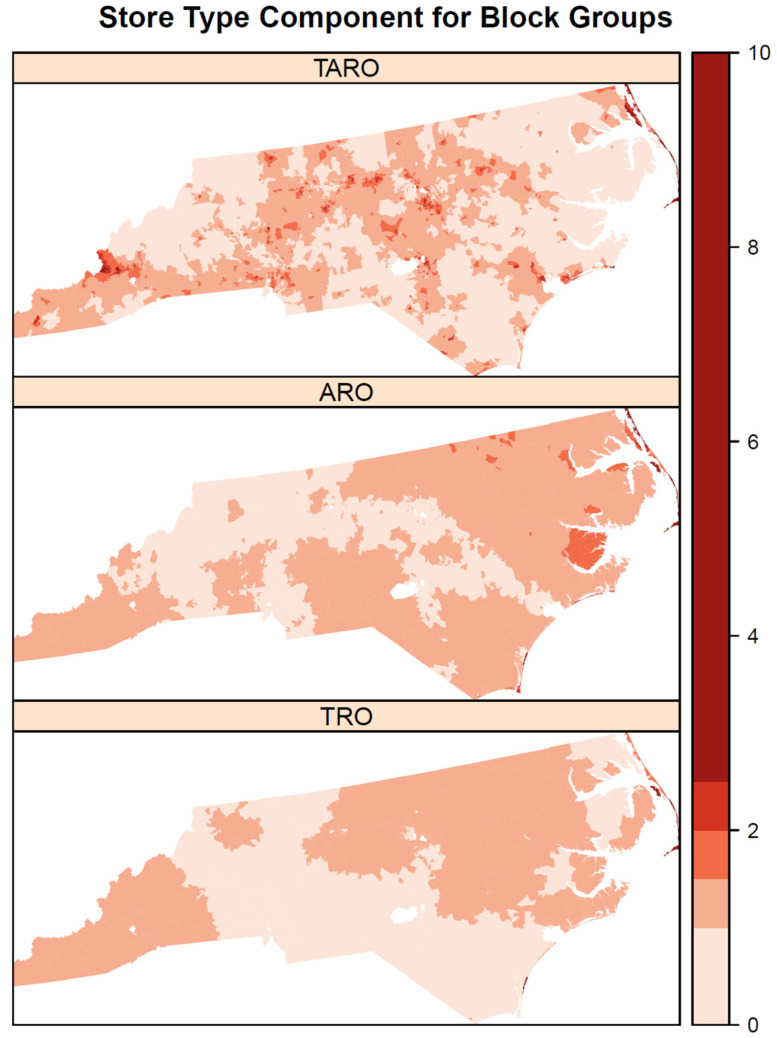
Outlet (TRO, ARO, and TARO) specific spatial component for block groups.

The shared spatial component of relative risk (Figure 6) shows some spatial heterogeneity with high values scattered throughout the state but with a concentration of high values along the eastern coast. In contrast, there are some clusters of low shared risk for the three outlet types in western portions of the state. The outlet-specific components (Figure 7) show more spatial homogeneity for AROs and TROs with large swaths of elevated risk in southwestern and northeastern North Carolina. Compared with AROs and TROs, the spatial component for TAROs has more spatial heterogeneity with many small clusters of elevated risk in central and southwestern North Carolina.

## 4. Discussion

In this paper, we aimed to estimate a neighborhood disadvantage index to explain variation in rates for TROs, AROs, and TAROs using a novel Bayesian shared component index model. We found significant positive associations between neighborhood disadvantage and the rates of each outlet type, but the association was greatest for outlets selling both tobacco and alcohol (i.e., TAROs). The most important variables in explaining these associations were percent renters (i.e., low home ownership), percent of homes built before 1940 (i.e., old housing stock), and percent without a high school diploma (i.e., low education). Rates were positively correlated among the three outlet types and there was a shared component to explain relative risks for the three types of outlets. All three outlet types had significantly elevated risk in the northeastern shore area of the Outer Banks, meaning that more outlets were observed in this area than expected given the overall rates in the state. In addition, all three outlet types had spatial heterogeneity in relative risk, indicating geographic disparities in the neighborhood burden of outlets selling one or both of alcohol and tobacco. However, there were also notable differences in the patterns of relative risks by outlet type, where TROs and AROs had larger clusters of elevated risk in the northeast and southeastern coast compared with TAROs, and AROs in particular had a notable cluster of elevated risk in northeastern North Carolina along the border of Virginia. In fact, the clearest disparities in relative risk were for AROs, with high values in the northeast and low values elsewhere in the state. In addition, relative risk was highest in suburban areas compared with urban and rural areas for all three outlet types.

Overall, the findings in our study build upon previous literature examining neighborhood deprivation by simultaneously considering the associations of TROs, AROs, and TAROs. Previous studies have focused on only TROs [15,25] or only AROs [10,26], with none examining the combination of these retail outlets, to the best of our knowledge. Other studies have examined rates of smoking and alcoholism in contexts outside of the United States, such as in North India [27], where both TROs and AROs were geocoded by physically walking in the community and using a map app to determine the coordinates of the outlets. Prevalence of smoking and drinking were associated with TRO density but not ARO density. In Scotland, Shortt and colleagues [11] examined both TROs and AROs and their associations with neighborhood deprivation, which, when compared to our study, similarly found that higher densities of these retail outlets are more prevalent in more deprived neighborhoods. However, neighborhood deprivation in that study was assessed only by income and by those needing government benefits and assistance. An additional study of TROs and AROs in central Massachusetts calculated the ratio of the densities of TROs to AROs in block groups by percentage minority population but did not directly consider businesses that sold both products [28]. Our study provides a novel contribution to the literature through its analysis of businesses that sell tobacco, alcohol, and both at the same time. Comparing these three outlet types and their associations with neighborhood characteristics allows for a more precise understanding of the types of businesses that vary in distribution in response to neighborhood composition. In this case, it led to the finding that TAROs have the strongest association with neighborhood deprivation when compared with TROs and AROs. 

In this study, we employed novel analytic methods to assess the spatial distribution of alcohol and tobacco outlets and the relationships with neighborhood sociodemographic characteristics. Using a Bayesian index regression model to estimate the NDI is an advantage compared with other dimension reduction techniques that have been used previously [29,30] such as principal component analysis (PCA) and factor analysis, which produce components that are difficult to interpret and are constructed independently of the association with the outcome. In contrast, our Bayesian index model estimates the index weights considering associations with the outcomes and produces easily interpretable relative importance weights, some of which can be effectively zero for unimportant variables. In our case, only three variables showed up as being relatively important while four had very small weights. We previously demonstrated that, using an index approach to simultaneously estimate the NDI and its health effect leads to significantly better model goodness-of-fit than using PCA to construct the index [31]. Another strength is the extension of the Bayesian index regression model to accommodate multiple outcomes with a shared component. We used the information in the three outlet types to estimate the neighborhood disadvantage index for all block groups together but allowed the effect of the NDI to differ for each outlet type, which, in this case, was useful, as shown by the significantly higher effect for outlets selling both tobacco and alcohol (TAROs).

The strengths of this study should be considered in light of the limitations. Notably, we focused on the association of outlet rates and neighborhood disadvantage as measured through certain sociodemographic variables, and did not consider other covariates at the block group level or other levels such as the county. In addition, while our study area included all of North Carolina, future research should apply the methods in this study to other geographic regions where norms around smoking and drinking may differ (e.g., norms that promote or discourage alcohol and tobacco use), as well as the policies regulating sales and zoning. Additionally, TRO and ARO data were drawn from large business registries and our TRO data relied on an algorithm for classifying individual stores as likely tobacco retailers. We performed checks to verify these listings to the degree possible, but some error attributable to input data is nonetheless expected (e.g., presence of unregistered businesses or those otherwise not included in the original listings). 

## 5. Conclusions

Our use of Bayesian shared component index models uncovered significant positive associations between neighborhood disadvantage and rates of tobacco, alcohol, and tobacco and alcohol outlets in North Carolina and identified clear geographic disparities in neighborhood burden of these outlets. The factors driving the significant associations include low levels of home ownership, old housing stock, and low education levels. These findings increase our understanding of neighborhood factors related to the unequal distribution of alcohol and tobacco outlets and therefore environmental factors related to smoke exposure and alcohol use. Further bolstering our understanding of both individual and environmental factors of smoke exposure remains a critical public health issue, especially considering the potential for adverse health outcomes in vulnerable groups such as newborns and children [32]. Future research endeavors should focus on addressing educational and economic disparities in disadvantaged areas, especially as they relate to smoking and alcohol use, as well as addressing the structural inequalities that influence smoking and alcohol use in these neighborhoods, which often have disproportionately higher numbers of racial and ethnic minorities.

## Figures and Tables

**Table 1:** Mean relative risks and percent significantly elevated relative risks for outlet types by population density category of block groups.

	Rural	Suburban	Urban
Relative Risk			
Tobacco	1.117	1.400	1.157
Alcohol	1.152	1.435	1.159
Tobacco and Alcohol	0.946	1.438	1.372
Percent Significant			
Tobacco	0.099	0.198	0.141
Alcohol	0.113	0.190	0.133
Tobacco and Alcohol	0.060	0.153	0.148

## Data Availability

Publicly available datasets were analyzed in this study. NETS data are available from Don Wells at dwalls2@earthlink.net. ARO data are available at https://abc.nc.gov/Permit/Retail (accessed on 19 January 2022). ACS data are available at https://www.census.gov/programs-surveys/acs (accessed on 19 January 2022).

## Supplementary Materials

The following are available online at https://www.mdpi.com/article/10.3390/ijerph19031134/s1, Figure S1: Gelman-Rubin convergence diagnostic plots for key model parameters.
