# Supplementary material for: Associations of Alcohol and Tobacco Retail Outlet Rates with Neighborhood Disadvantage
Source: Int J Environ Res Public Health. 2022 Jan 20;19(3):1134. doi: 10.3390/ijerph19031134 (PMC8834944; doi:10.3390/ijerph19031134)
Supplement: Supplementary file 1 [file ijerph-19-01134-s001.zip › ijerph-1520767-supplementary.pdf]

Supplemental Material

Associations of Alcohol and Tobacco Retail Outlet Rates with Neighborhood Disadvantage

David C. Wheeler [1,*], Joseph Boyle [1], D. Jeremy Barsell [2], Trevin Glasgow [2], F. Joseph McClernon [3], Jason A. Oliver [3,4,5] and Bernard F. Fuemmeler [2,6]

[1] Department of Biostatistics, Virginia Commonwealth University, Richmond, VA 23298, USA; boylejr@vcu.edu
[2] Department of Health Behavior and Policy, Virginia Commonwealth University, Richmond, VA 23298, USA; barselldtj@vcu.edu (D.J.B.); trevin.glasgow@vcuhealth.org (T.G.); Bernard.fuemmeler@vcuhealth.org (B.F.F.)
[3] Department of Psychiatry and Behavioral Sciences, Duke University School of Medicine, Durham, NC 27705, USA; francis.mcclernon@duke.edu (F.J.M.); jason-oliver@ouhsc.edu (J.A.O.)
[4] Stephenson Cancer Center, University of Oklahoma Health Sciences Center, Oklahoma City, OK 73104, USA
[5] Department of Psychiatry and Behavioral Sciences, Oklahoma State University Center for Health Sciences, Tulsa, OK 74107, USA
[6] Massey Cancer Center, Virginia Commonwealth University, Richmond, VA 23298, USA
* Correspondence: dcwheeler@vcu.edu; Tel.: +804-828-9827

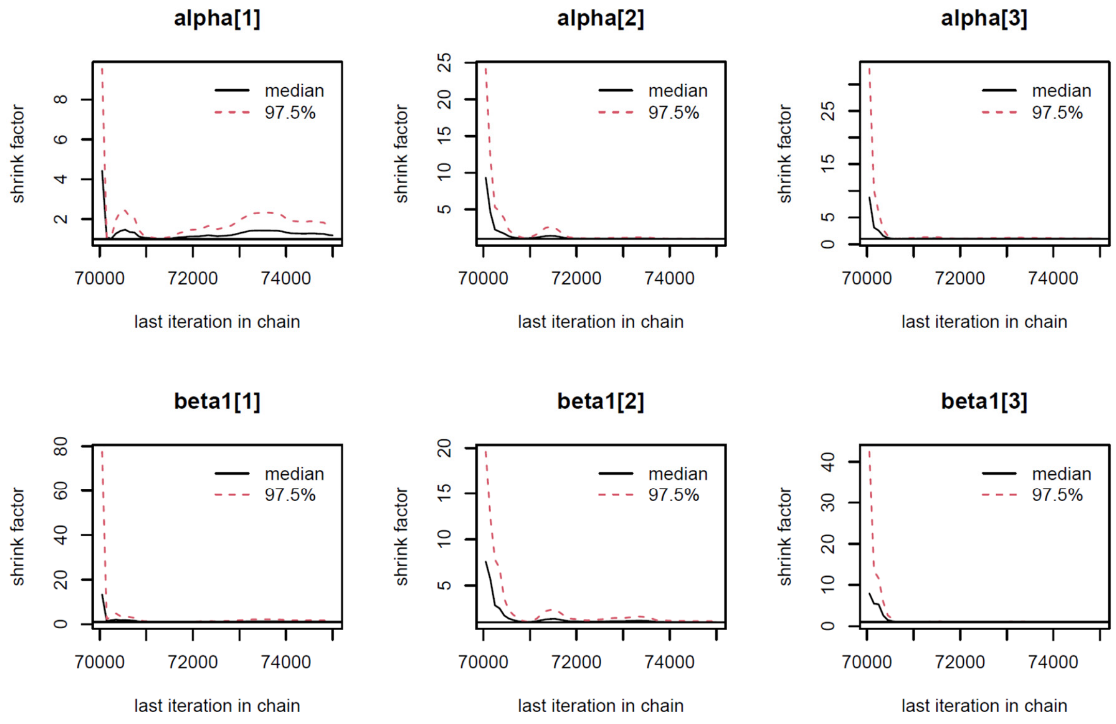

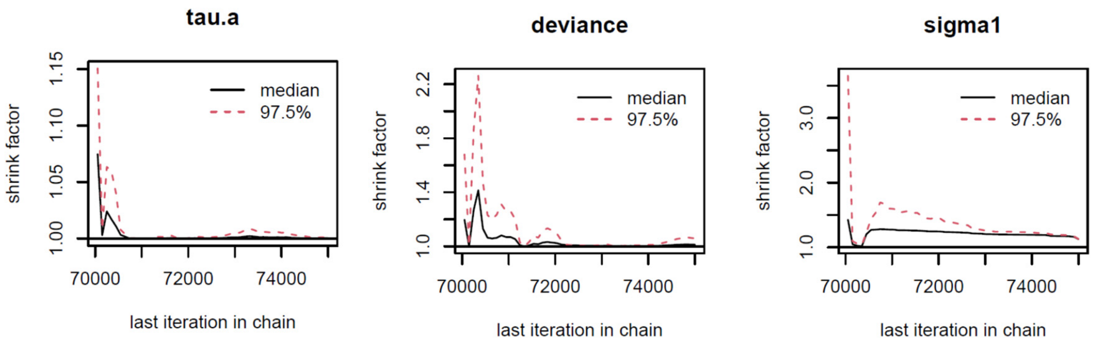

Figure S1. Gelman-Rubin convergence diagnostic plots for key model parameters.
